# Symmetry Evolution of La_2_O_3_ from P**$\overset{-}{3}$**m1 to P6_3_/mmc for Enhanced Electrocatalytic H_2_O_2_ Production

**DOI:** 10.3390/nano16080469

**Published:** 2026-04-15

**Authors:** Hansong Yuan, Yuheng Gu, Qian Yang, Shun Li, Jianming Zhang, Long Zhang, Yuqiao Zhang

**Affiliations:** School of Chemistry and Chemical Engineering, Jiangsu University, Zhenjiang 212013, China; yhs0201@stmail.ujs.edu.cn (H.Y.); gyh20010301@stmail.ujs.edu.cn (Y.G.); shun@ujs.edu.cn (S.L.); zhangjm@ujs.edu.cn (J.Z.); longzhang@ujs.edu.cn (L.Z.)

**Keywords:** electrocatalysis, La_2_O_3_, SrTiO_3_, two-electron ORR, H_2_O_2_ synthesis

## Abstract

Electrocatalytic H_2_O_2_ production via the two-electron oxygen reduction reaction (ORR) is a highly sustainable alternative to industrial methods. To further optimize non-noble catalysts, we report an interfacial engineering strategy to stabilize the metastable P6_3_/mmc-La_2_O_3_ phase on SrTiO_3_. This symmetry evolution from the low-symmetry $P\overset{-}{3}m1$ (trigonal) to the high-symmetry P6_3_/mmc (hexagonal) space group yields a composite with >95% H_2_O_2_ selectivity. Mechanistic studies demonstrate that the symmetry-regulated interface optimizes *OOH conversion and suppresses O–O bond cleavage. This work offers a robust design principle for high-performance, noble-metal-free H_2_O_2_ electrosynthesis.

## 1. Introduction

Hydrogen peroxide (H_2_O_2_) is a versatile and industrially important oxidant that has been widely used in fields such as textile bleaching, wastewater treatment, chemical synthesis, and biological disinfection [1,2,3,4]. To date, the anthraquinone process remains the dominant industrial route for large-scale H_2_O_2_ production. However, this method involves multistep hydrogenation/oxidation cycles and extensive solvent management, which translate into energy-intensive operation, a complex reaction flow, and non-negligible environmental burdens [5,6]. In contrast, electrocatalytic synthesis has emerged as a promising alternative owing to its low energy requirements, mild reaction conditions, and minimal environmental impact. Among these, the two-electron oxygen reduction reaction (ORR) provides a sustainable and environmentally friendly pathway to generate H_2_O_2_ under mild conditions [7,8,9,10].

One of the major challenges of the ORR lies in controlling the selectivity between two competing reaction pathways: the four-electron pathway to H_2_O and the two-electron pathway to H_2_O_2_ [8,11,12]. An effective two-electron ORR catalyst requires intermediate oxygen binding, which is sufficient to enable O_2_ activation and *OOH formation, while avoiding excessive adsorption that drives O–O bond scission and the four-electron pathway. Noble metals such as platinum (Pt) and palladium (Pd) typically exhibit high O–O bond dissociation ability, thus favoring the four-electron pathway and resulting in poor H_2_O_2_ selectivity [13,14,15,16]. Therefore, the development of cost-effective, stable, and highly selective non-noble metal catalysts for the two-electron ORR is of great significance [12,17,18].

Rare-earth oxides and perovskite-type oxides have been increasingly explored as electrocatalysts, because their electronic structures and defect chemistry can be engineered over a wide range, enabling tailored surface reactivity [19,20,21,22]. Rare earth oxides, especially La_2_O_3_, have been extensively studied in heterogeneous catalysis due to their tunable electronic structures [23]. However, the influence of crystal symmetry on their electrocatalytic behavior remains insufficiently explored. La_2_O_3_ can exist in various polymorphs with different space groups, such as $P\overset{-}{3}m1$ and P6_3_/mmc, which differ in atomic arrangement, coordination environment, and symmetry operation. This structural variation may affect the distribution of surface electronic states and the uniformity of active sites, thereby influencing the adsorption configuration of reaction intermediates. Nevertheless, the relationship between La_2_O_3_ symmetry evolution and the pathway selectivity of ORR, particularly for H_2_O_2_ formation, has not been systematically investigated. Perovskite oxides (e.g., SrTiO_3_) have been extensively studied as electrocatalysts due to their structural stability and tunable electronic properties. SrTiO_3_ can provide efficient electron transport channels and stable catalyst supports [24,25]. However, due to the imperfect binding of oxygen intermediates, the intrinsic activity of SrTiO_3_ for the selective 2e^−^ ORR is generally limited. On the other hand, although La_2_O_3_ exhibits good adsorption properties for oxygen-containing species, its relatively low conductivity may hinder effective charge transfer in the electrocatalytic process. Constructing a composite system that integrates La_2_O_3_ and SrTiO_3_ therefore presents a promising strategy to combine their complementary advantages. More importantly, the formation of the La_2_O_3_/SrTiO_3_ heterointerface may induce electronic structure redistribution, thereby optimizing the adsorption strength of the *OOH intermediate and improving the selectivity for H_2_O_2_. Furthermore, the SrTiO_3_ matrix may serve as a structural template to stabilize specific polymorphs of La_2_O_3_, enabling symmetry evolution that is otherwise difficult to achieve under conventional conditions. However, the interaction mechanisms between interfacial interactions, symmetry evolution, and catalytic selectivity in such composite systems remain unclear.

In this study, the metastable phase of hexagonal La_2_O_3_ with P6_3_/mmc symmetry has been stabilized and grown on SrTiO_3_ template powders via a conventional solid-state reaction method. By tuning La_2_O_3_ content, we observe a composition-dependent symmetry evolution of the hexagonal La_2_O_3_ phase, where the phase assignment shifts from $P\overset{-}{3}m1$ to P6_3_/mmc. This enhanced symmetry thus improved the uniformity of surface electronic distribution. Electrochemical measurements demonstrated that, compared with pure La_2_O_3_ with $P\overset{-}{3}m1$ symmetry and SrTiO_3_, the optimized La_2_O_3_/SrTiO_3_ composite catalyst delivers significantly higher H_2_O_2_ yield and two-electron selectivity. These results highlight the pivotal role of crystallographic symmetry evolution in steering the ORR toward the two-electron pathway. This study provides new insights into the role of crystallographic symmetry and interfacial synergy in regulating ORR selectivity.

## 2. Materials and Methods

### 2.1. Catalyst Synthesis

**Figure 1:**
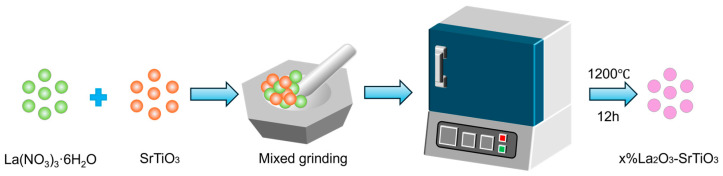
Schematic illustration of the synthesis procedure of La_2_O_3_-SrTiO_3_ composite electrocatalysts.

The schematic representation of the synthesis of Catalyst Synthesis is given in Figure 1. In this study, La_2_O_3_-SrTiO_3_ composite electrocatalysts with different La_2_O_3_ content were synthesized via a conventional high-temperature solid-state reaction route. Specifically, SrTiO_3_ and La(NO_3_)_3_·6H_2_O powders were weighed to match the desired molar ratios between La_2_O_3_ and SrTiO_3_, followed by thorough mixing and grinding for 1 h. The resulting mixture was heated to 1200 °C in air at a heating rate of 10 °C/min and calcined for 12 h to ensure complete decomposition of the nitrate precursor and formation of crystalline La_2_O_3_. At the same time, the high-temperature treatment promoted the interfacial interaction between La_2_O_3_ and SrTiO_3_, which helped La_2_O_3_ to partially transform from the thermodynamically stable $P\overset{-}{3}m1$ phase to the metastable P6_3_/mmc phase, thereby obtaining La_2_O_3_-SrTiO_3_ electrocatalysts with different La_2_O_3_ contents. The obtained samples are denoted as *x*%La_2_O_3_-SrTiO_3_ (*x* = 1, 3, 5, 10, 15, and 20) according to the nominal La_2_O_3_ content, where *x* represents the molar percentage of La_2_O_3_ relative to SrTiO_3_. For example, the 10% La_2_O_3_–SrTiO_3_ sample corresponds to a molar ratio of La_2_O_3_:SrTiO_3_ = 1:9. For comparison, pure La_2_O_3_ powders were yielded by annealing La(NO_3_)_3_·6H_2_O powder under the same conditions.

### 2.2. Physical Characterization

The phase composition of the samples was analyzed by X-ray diffraction (XRD, SmartLab, Rigaku, Tokyo, Japan). The morphology of the catalysts was examined by cold-field emission scanning electron microscopy (SEM, SU8600, Hitachi, Tokyo, Japan) and transmission electron microscopy (TEM, JEM-2100, JEOL Co., Ltd., Tokyo, Japan). X-ray photoelectron spectroscopy (XPS, Al Kα, ESCALAB QXi, Thermo Fisher Inc., Waltham, MA, USA) was employed to analyze the surface chemical composition of the electrocatalysts. In situ Fourier transform infrared spectroscopy (FTIR, Nicolet iS50 FT-IR, Thermo Fisher Scientific Inc., Waltham, MA, USA) and high-resolution mass spectrometry (HRMS, Q Extractive plus, Thermo Fisher Scientific Inc., Waltham, MA, USA) were employed to monitor the formation and evolution of surface reaction intermediates during the oxygen reduction reaction (ORR) process, thereby elucidating the reaction pathway and the underlying mechanism for H_2_O_2_ generation.

### 2.3. Electrochemical Measurements

Electrochemical measurements were carried out using an electrochemical workstation (CHI760E, CH Instruments, Shanghai, China) coupled with a rotating ring-disk electrode (RRDE) system (AFMSRCE type, Pine Instruments Corporation, Durham, NC, USA). Prior to the measurements, catalyst inks were prepared as follows: 5 mg of the as-synthesized catalyst powder was dispersed in 1 mL of anhydrous ethanol and mixed with 10 µL of Nafion solution (5 wt%, DuPont, Wilmington, DE, USA). The mixture was ultrasonicated for 30 min to form a homogeneous suspension.

When conducting tests using an electrochemical workstation, different doses of catalyst ink were taken and dropped onto an indium tin oxide (ITO) conductive glass fixed with a rubber sealing ring using a pipette. The ink was then dried under an infrared lamp and used as the working electrode. Ag/AgCl was used as the reference electrode, and a Pt mesh was used as the counter electrode. Based on this, the electrochemical behavior under different reaction conditions in 0.1 M KCl solution was studied, and the yield of H_2_O_2_ in the solution after each reaction was determined using the iodometric titration method.

In RRDE experiments, the geometric areas of the ring and disk electrodes were 0.2475 cm^2^ and 0.1866 cm^2^, respectively. A 10 µL aliquot of the catalyst ink was drop-cast onto the disk electrode and allowed to dry naturally, forming a uniform catalytic film. The Ag/AgCl electrode and a Pt mesh were used as the reference and counter electrodes, respectively. The reference electrode potential was calibrated to the reversible hydrogen electrode (RHE) using the following equation:

*E* (*V* vs. RHE) = *E* (*V* vs. Ag/AgCl) + *E*^Θ^(Ag/AgCl) + 0.059 × pH

### 2.4. Theoretical Calculations

We used the DFT as implemented in the Vienna Ab initio simulation package (VASP) in all calculations. The exchange-correlation potential is described by using the generalized gradient approximation of Perdew–Burke–Ernzerhof (GGA-PBE). The projector augmented-wave (PAW) method is employed to treat interactions between ion cores and valence electrons. The plane-wave cutoff energy was fixed to 450 eV. Given structural models were relaxed until the Hellmann–Feynman forces were smaller than −0.02 eV/Å and the change in energy was smaller than 105 eV. Grimme’s DFT-D3 methodology was used to describe the dispersion interactions among all the atoms in adsorption models.

The Gibbs free energy change is defined as:

Δ*G* = Δ*E* + Δ*ZPE* − *T*Δ*S*

where Δ*E* is the electronic energy calculated with VASP, Δ*ZPE* and Δ*S* are the zero-point energy difference and the entropy change between the products and reactants, respectively, and T is the temperature (298.15 K).

## 3. Results and Discussion

**Figure 2:**
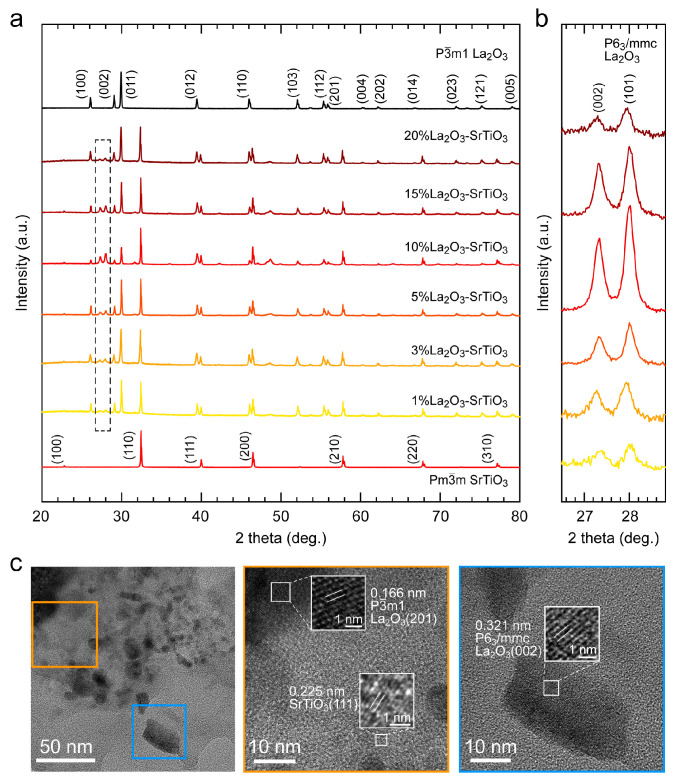
Structural characterization of La_2_O_3_-SrTiO_3_ composite powders. (**a**) XRD patterns of La_2_O_3_-SrTiO_3_ composite powders with different La_2_O_3_ fractions. (**b**) Magnified XRD patterns of the dashed box in (**a**). (**c**) TEM images of 10% La_2_O_3_-SrTiO_3_ sample.

As illustrated by the X-ray diffraction (XRD, SmartLab, Rigaku) patterns in Figure 2a,b, annealing the mixture of La(NO_3_)_3_·6H_2_O and SrTiO_3_ effectively induces the formation of a La_2_O_3_/SrTiO_3_ heterostructure. The diffraction peaks of La_2_O_3_ can be indexed to two phases with different symmetries. For the $P\overset{-}{3}m1$ phase, the main characteristic peaks are observed at 2θ ≈ 26.08°, 29.08°, 29.92°, 39.46°, 46.02°, 52.1°, 55.38° and 55.9°, corresponding to the (100), (002), (011), (012), (110), (103), (112) and (201) planes. The appearance of diffraction peaks at 2θ ≈ 27.42° and 28.11° can be assigned to the (002) and (101) planes of the P6_3_/mmc phase. With increasing La_2_O_3_ fraction, the relative contribution of the P6_3_/mmc phase exhibits a non-monotonic dependence, reaching its peak at *x* = 10% and decreasing at higher La_2_O_3_ loading. It is worth noting that the $P\overset{-}{3}m1$ is considered the most thermodynamically favored polymorph of La_2_O_3_ under ambient conditions, whereas formation of the metastable P6_3_/mmc phase typically occurs only at temperatures exceeding 2000 °C [26]. Moreover, annealing pure La(NO_3_)_3_·6H_2_O powder exclusively yields the $P\overset{-}{3}m1$ phase. Consequently, the appearance of the P6_3_/mmc phase in the composites can be attributed to the SrTiO_3_ matrix acting as a structural template. Such stabilization may arise from the epitaxial strain originating from the SrTiO_3_ lattice, which could lower the energetic penalty for forming the metastable P6_3_/mmc phase. To further elucidate the phase distribution and interfacial structure between the La_2_O_3_ and SrTiO_3_ phases, transmission electron microscopy (TEM, JEM-2100, JEOL Co., Ltd.) analysis was performed on the representative 10%La_2_O_3_-SrTiO_3_ sample. As shown in Figure 2c, it shows lattice stripes of 0.166 nm, 0.225 nm and 0.321 nm, which belong to the (201) crystal plane of La_2_O_3_ ($P\overset{-}{3}m1$), the (111) crystal plane of SrTiO_3_ and the (002) crystal plane of La_2_O_3_ (P6_3_/mmc), respectively. By combining the EDS plot (Figure S1 and Table S1), we can see that the elements are uniformly distributed. It is inferred that La_2_O_3_ domains (in both $P\overset{-}{3}m1$ and P6_3_/mmc phases) with the particle size of 10~30 nm are well dispersed on the surface of the SrTiO_3_ matrix. It is hypothesized that during the initial stage of annealing, the decomposition of La(NO_3_)_3_·6H_2_O releases acidic gaseous species, which may mildly etch the SrTiO_3_ surface and promote subsequent La_2_O_3_ nucleation [27,28]. This process could create additional active sites, enabling more uniform dispersion of La_2_O_3_ upon high-temperature conversion of the precursor [29]. As a result, a uniform distribution of La_2_O_3_ is achieved, promoting efficient contact between the active species and the catalyst during electrocatalysis. X-ray photoelectron spectroscopy (XPS) results showed that the La 3d spectrum exhibited characteristic peaks corresponding to La^3+^, confirming the presence of La–O bonds in La_2_O_3_. The Ti 2p spectrum showed two main peaks belonging to Ti^4+^, indicating the presence of Ti–O bonds in the SrTiO_3_ lattice. While high concentrations of oxygen vacancies and La doping would induce Ti^3+^ formation [25,30], no Ti^3+^ was observed in this study, suggesting a negligible donor-doping effect from either La^3+^ or oxygen vacancy. This further indicates that the compositing between La_2_O_3_ and SrTiO_3_ did not introduce a donor doping from La^3+^ to the Ti 3d orbital, where only Ti 2p peaks attributed to Ti^4+^ can be observed (Figure S2). Therefore, these observations support the formation of a surface-decorated La_2_O_3_/SrTiO_3_ composite, where La_2_O_3_ is primarily located or near the SrTiO_3_ surface rather than being incorporated into the SrTiO_3_ lattice.

When comparing the catalytic performance of catalysts, differences in electrochemically active surface area (ECSA) must be considered to more accurately assess the surface effect on ORR activity. Bilayer capacitance (*C*_dl_) is used to estimate the corresponding ECSA differences. Bilayer capacitance is determined by *CV* curves using the following equation:

Δ*j* = (*j*_a_ − *j*_c_)/2 = *C*_dl_ × *v* (1)

In the charging current density plot, the scan rate (*v*) is plotted on the *x*-axis and Δ*j* on the *y*-axis, where *j*_a_ and *j*_c_ represent the anode and cathode current densities at Δ*E* = 0 V (potential window was 0.927 V to 1.027 V vs. RHE), respectively, and v represents the scan rate in mV s^−1^. The slope of the plot represents the electric double-layer capacitance (*C*_dl_). The non-Faradic current density based on the ECSA was then estimated using the following equation:

ECSA = *C*_dl_/*C*_s_ (2)

where *C*_s_ represented the specific capacitance of the electrode. The corresponding CV curves and the difference between the charging current density Δ*j* and the scan rate (*v*) are shown in Figure S3. The *C*_dl_ values of different catalysts are shown in Table S2. Compared to SrTiO_3_, the *C*_dl_ values of all catalysts increased, with the 10% La_2_O_3_-SrTiO_3_ composite exhibiting the highest *C*_dl_ value, indicating that it possesses the largest electrochemically active surface area and a higher density of active sites. These results demonstrate that the introduction of La_2_O_3_ can effectively increase the exposure of catalytic sites and improve the electrochemical interface.

Furthermore, we conducted stability tests using chronoamperometry in an oxygen-rich KCl electrolyte at a reaction potential of 0 V (vs. RHE) to evaluate the catalyst’s durability. As shown in Figure S4, the current density of the 10% La_2_O_3_-SrTiO_3_ electrocatalyst remained relatively stable during the test, indicating that the composite catalyst has good electrochemical stability.

**Figure 3:**
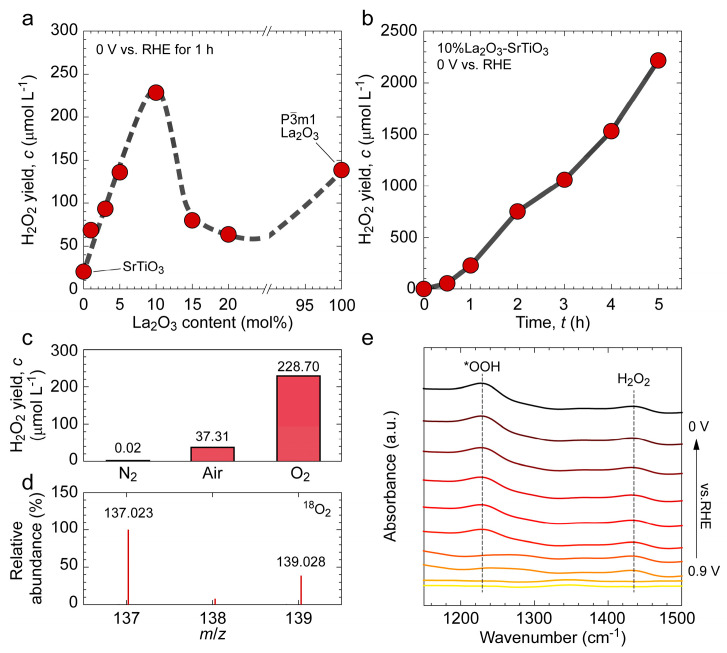
Electrocatalytic H_2_O_2_ production performance and mechanistic evidence for La_2_O_3_-SrTiO_3_ composite. (**a**) La_2_O_3_ content-dependent H_2_O_2_ yield of La_2_O_3_-SrTiO_3_ composite powders under the O_2_ saturation condition. (**b**) Time-dependent H_2_O_2_ yield over an optimized 10%La_2_O_3_-SrTiO_3_ sample under the O_2_ saturation condition. (**c**) H_2_O_2_ yields over a 10%La_2_O_3_-SrTiO_3_ sample under different atmospheres. (**d**) LC-MS results of TECatal reaction by ^18^O isotope labeling. (**e**) In situ FTIR spectroscopy of a 10% La2O3-SrTiO3 sample at different applied voltages.

The H_2_O_2_ electrosynthesis performance of La_2_O_3_-SrTiO_3_ catalysts with different La_2_O_3_ content was evaluated at 0 V vs. reversible hydrogen electrode (RHE) using a standard three-electrode configuration, and the results are summarized in Figure 3. Figure 3a shows that with a fixed catalyst loading of 0.5 mg and electrolysis time of 1 h, the produced concentration of H_2_O_2_ follows a clear volcano-type trend with increasing La_2_O_3_ content. A maximum H_2_O_2_ yield of 228.70 μmol L^−1^ was obtained in the 10%La_2_O_3_-SrTiO_3_ sample. Notably, this volcano-like tendency is well consistent with the evolution of the P6_3_/mmc La_2_O_3_ phase (Figure 2c), suggesting that the presence of the P6_3_/mmc polymorph and its interface with SrTiO_3_ contributes positively to H_2_O_2_ formation. Meanwhile, the pure La_2_O_3_ with $P\overset{-}{3}m1$ symmetry displays a lower H_2_O_2_ yield of 138.64 μmol L^−1^, suggesting that the enhanced performance of the composite cannot be explained by La_2_O_3_ alone and is instead linked to the symmetry-modulated La_2_O_3_ phase and interfacial effect. In the case of the 10%La_2_O_3_-SrTiO_3_ sample, we further tested the time-dependent electrocatalytic performance. As shown in Figure 3b, prolonging the electrolysis to 5 h leads to a constant increase in H_2_O_2_ production without any decay of the reaction rate, indicating a robust catalytic activity over the tested duration. The generation of H_2_O_2_ usually involves two pathways, which are water oxidation reaction (WOR) and ORR. In order to clarify the reaction pathway in this study, we compared the electrocatalytic performance under different atmospheres (N_2_, air, and O_2_). As shown in Figure 3c, H_2_O_2_ formation is essentially suppressed under N_2_, whereas it is significantly enhanced under O_2_, with air giving an intermediate level. Therefore, these atmosphere-resolved results indicate that dissolved O2 is required for H_2_O_2_ formation, supporting ORR as the dominant pathway under the applied conditions. In addition, further evidence is provided by the ^18^O_2_ isotope labeling experiment using 4-hydroxybenzoic acid (4-HPBA) as a molecular probe to trace the oxygen source in the generated H_2_O_2_. The emergence of a distinct signal at *m*/*z* = 139.028 in the high-resolution mass spectrum confirms incorporation of oxygen from O_2_ into the generated H_2_O_2_ (Figure 3d) [31]. To further probe the evolution of reaction intermediates and the reaction mechanism of the La_2_O_3_-SrTiO_3_ electrocatalyst during the ORR process, in situ Fourier-transform infrared (FTIR) spectroscopy (Nicolet iS50 FT-IR, Thermo Fisher Scientific Inc.) was performed on the 10%La_2_O_3_-SrTiO_3_ sample in 0.1 M KCl solution. Figure 3e presents the FTIR spectra recorded at various applied potentials (vs. RHE). As the applied potential was gradually shifted to more negative values, an absorption band emerged and gradually intensified at approximately 1230 cm^−1^ from 0.7 V, which can be assigned to the O–O stretching vibration of the hydroperoxyl (*OOH) intermediate during the reaction. In addition, the signals observed in the range of 1400–1450 cm^−1^ are attributed to the vibrational features of H_2_O_2_.

Based on the results above, a plausible reaction pathway involving a two-electron reduction mechanism has been deduced [11]:

O_2_ + H^+^ + *e*^−^ → *OOH

     *OOH + H^+^ + *e*^−^ → H_2_O_2_

To further reveal the interfacial charge transport behavior of the La_2_O_3_-SrTiO_3_ composite material, electrochemical impedance spectroscopy (EIS) was performed on different La_2_O_3_-SrTiO_3_ composite material samples (Figure S5). The charge transfer resistance (*R*_1_) was simulated using the equivalent circuit model shown in Figure S6, and the results are presented in Table S3. The composite catalyst with 10% La_2_O_3_ content exhibited the lowest *R*_1_ values, indicating that this sample not only has the lowest bulk resistance but also the most rapid interfacial charge transfer process. This suggests that the introduction of an appropriate amount of La_2_O_3_ helps to construct a more efficient electron transport network and significantly reduces the energy barrier during the electrode reaction process.

**Figure 4:**
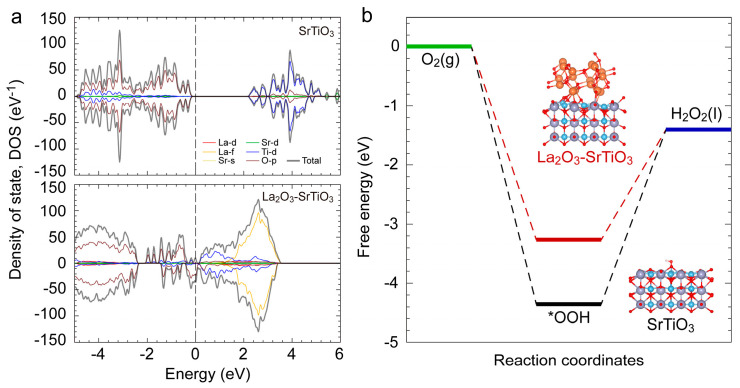
Theoretical investigation of the two-electron ORR mechanism. (**a**) Total and partial electronic density of states (DOS) for the catalysts. (**b**) Gibbs free energy profiles for the two-electron ORR pathway on pristine SrTiO_3_ and La_2_O_3_-SrTiO_3_ composite surfaces. The insets display the optimized adsorption configurations of reaction intermediates.

To gain an in-depth understanding of the two-electron ORR pathway and catalytic mechanism of the La_2_O_3_-SrTiO_3_ composite catalyst, density functional theory (DFT) calculations were further performed to analyze the electronic structure and the energetics of key reaction steps. Figure 4a presents the calculated electronic density of states (DOS) for SrTiO_3_ and La_2_O_3_-SrTiO_3_ composite model. The composite shows an increased density of electronic states near the Fermi level relative to pristine SrTiO_3_, resulting from interfacial electronic reconstruction. Such a feature is expected to improve the electrical conductivity of the catalyst and thereby facilitate charge transfer during the electrocatalytic process. Moreover, the computed free energy profiles shown in Figure 4b reveal that pristine SrTiO_3_ suffers from a large uphill free energy change for the rate-determining step (*OOH + H^+^ + *e*^−^ → H_2_O_2_), with a Δ*G* value of approximately 2.95 eV, indicating its unfavorable energetics to further activate and convert the *OOH intermediate. In contrast, this uphill free-energy requirement is significantly reduced in the La_2_O_3_-SrTiO_3_ composite structure, with a Δ*G* value of approximately 1.87 eV, demonstrating that the composite interface effectively lowers the energetic barrier associated with H_2_O_2_ formation. These DFT results are in good agreement with the experimentally observed enhancement in H_2_O_2_ generation, supporting that interfacial coupling in La_2_O_3_-SrTiO_3_ composite electrocatalyst can tune the adsorption energetics of oxygenated intermediates and lower the thermodynamic penalty for H_2_O_2_-forming steps in the two-electron ORR pathway.

**Figure 5:**
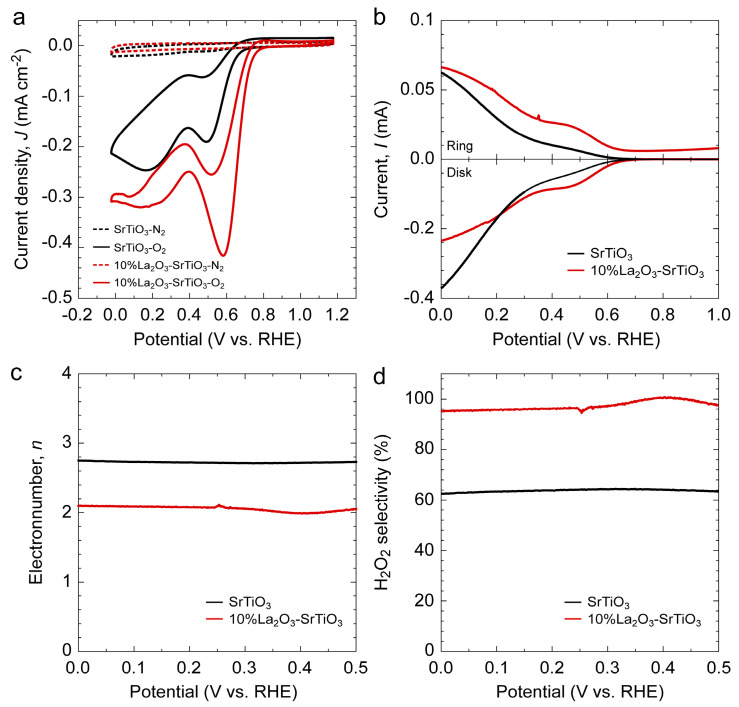
Selectivity of ORR in H_2_O_2_ production by La_2_O_3_-SrTiO_3_ composite powders. (**a**) Cyclic voltammogram (CV) curves, (**b**) disk and ring currents at 1600 rpm, (**c**) electron transfer number, and (**d**) H_2_O_2_ selectivity calculated from rotating ring–disk electrode (RRDE) responses for pure SrTiO_3_ and 10%La_2_O_3_-SrTiO_3_ powders.

In the electrocatalytic H_2_O_2_ production process, driving ORR toward a two-electron pathway is essential for efficient H_2_O_2_ generation. However, the competing four-electron reduction to H_2_O often occurs on many oxide surfaces, making selectivity control a key criterion for catalyst design [32,33]. To quantify the two-electron ORR selectivity, rotating ring–disk electrode (RRDE) measurements were conducted in a conventional three-electrode system using the 10%La_2_O_3_-SrTiO_3_ and pure SrTiO_3_ as catalysts. All tests of the ring-disk electrode were performed in 0.1 M KOH electrolyte. As shown in Figure 5a, cyclic voltammetry (CV) curves were recorded in N_2_ and O_2_-saturated electrolytes to confirm ORR activity. Both catalysts exhibit negligible reduction peaks in N_2_-saturated solution, whereas distinct reduction peaks appear under O_2_-saturated conditions. Compared to SrTiO_3_, the 10%La_2_O_3_-SrTiO_3_ sample displays a higher reduction current and a more positive onset potential, indicating its superior ORR activity. Further evaluation was performed using linear sweep voltammetry (LSV) under different rotation rates for the 10%La_2_O_3_-SrTiO_3_ sample, where the limiting diffusion current increases progressively with increasing rotation speed due to the enhanced mass transport and the reduced thickness of the diffusion layer (Figure S7) [34]. At a rotation rate of 1600 rpm, both disk and ring currents of 10%La_2_O_3_-SrTiO_3_ and SrTiO_3_ samples were recorded, as shown in Figure 5b. The 10%La_2_O_3_-SrTiO_3_ sample delivers higher disk and ring currents than SrTiO_3_, suggesting accelerated ORR kinetics and improved H_2_O_2_ formation. Based on the RRDE data, the H_2_O_2_ selectivity and electron transfer number (*n*) were calculated according to the following equations [35,36]:

(3) $$n\;=4\;\cdot \;\frac{I_{\mathrm{Disk}}}{I_{\mathrm{Disk}}+I_{\mathrm{Ring}}/N}$$

(4) $$H_{2}O_{2}(\%)\;=200\;\cdot \;\frac{I_{\mathrm{Ring}}/N}{I_{\mathrm{Disk}}+I_{\mathrm{Ring}}/N}$$

where *I*_Disk_ is the disk current, *I*_Ring_ is the ring current, and *N* is the collection efficiency, which is 0.37 in this system. As illustrated in Figure 5c, the *n* value for the 10%La_2_O_3_-SrTiO_3_ is close to 2, indicating a predominant two-electron ORR pathway for H_2_O_2_ production. Correspondingly, the H_2_O_2_ selectivity of 10%La_2_O_3_-SrTiO_3_ exceeds 95% across the tested potentials (Figure 5d), whereas SrTiO_3_ remains below 70%. As a result, compositing La_2_O_3_ with P6_3_/mmc symmetry into SrTiO_3_ brings a significant enhancement in the two-electron pathway, which improves the H_2_O_2_ production rate.

## 4. Conclusions

In this study, a series of La_2_O_3_-SrTiO_3_ composite electrocatalysts with different La_2_O_3_ content were successfully synthesized via a solid-state method. The interaction between La_2_O_3_ and SrTiO_3_ leads to discernible electronic modulation, which in turn governs the surface reactivity toward oxygen reduction. Specifically, a metastable hexagonal La_2_O_3_ phase with P6_3_/mmc symmetry can be stabilized in the presence of SrTiO_3_, which is typically inaccessible under conventional conditions. The evolution from a low-symmetry hexagonal phase (space group $P\overset{-}{3}m1$) to a high-symmetry hexagonal phase (space group P6_3_/mmc) correlates with improved H_2_O_2_ formation activity and selectivity, which we attribute to more favorable interfacial electronic structures and optimized adsorption energetics of *OOH intermediates. Furthermore, RRDE measurements confirm that the P6_3_/mmc symmetry La_2_O_3_ significantly enhances the selectivity toward the two-electron ORR pathway, resulting in a high H_2_O_2_ selectivity of over 95%. These findings not only highlight the importance of structural symmetry in modulating oxygen reduction pathways but also demonstrate a robust “template-induction” strategy to stabilize metastable rare-earth oxides. This work provides a new paradigm for the rational design of high-performance, cost-effective non-noble metal catalysts for decentralized and efficient H_2_O_2_ electrosynthesis.

## Data Availability

The data that support the findings of this study are available from the corresponding author upon reasonable request.

## Supplementary Materials

The following supporting information can be downloaded at https://www.mdpi.com/article/10.3390/nano16080469/s1, Figure S1: Mapping diagram of 10%La_2_O_3_-SrTiO_3_ sample; Table S1: The atomic fraction of each element; Figure S2: High-resolution X-ray photoelectron spectroscopy (XPS) spectra of (a) La 3d and (b) Sr 3d and (c) Ti 2p and (d) O 1s (O_C_ and O_L_ denote the surface-adsorbed oxygen and lattice oxygen, respectively); Figure S3: (a–g) Cyclic voltammetry curves of different electrocatalysts. (h) The corresponding electrochemically active surface area (ECSA) diagram; Table S2: Bilayer capacitance (*C*_dl_) of different catalysts; Figure S4: Stability testing of 10% La_2_O_3_-SrTiO_3_ electrocatalyst; Figure S5: Nyquist plots of different La_2_O_3_-SrTiO_3_ composite electrocatalysts; Figure S6: Equivalent circuit diagram for electrochemical impedance spectroscopy (EIS); Table S3: Electrochemical impedance spectroscopy fitting data; Figure S7: Linear sweep of voltammetry curves of the 10% La_2_O_3_-SrTiO_3_ sample at different rotation speeds.
