# Supplementary material for: Symmetry Evolution of La2O3 from P3 - m1 to P63/mmc for Enhanced Electrocatalytic H2O2 Production
Source: Nanomaterials (Basel). 2026 Apr 15;16(8):469. doi: 10.3390/nano16080469 (PMC13119269; doi:10.3390/nano16080469)
Supplement: Supplementary file 1 [file nanomaterials-16-00469-s001.zip › nanomaterials-4206966-supplementary.pdf]

**Supporting Information**

# Symmetry Evolution of $La_2O_3$ from $P\bar{3}m1$ to $P6_3/mmc$ for Enhanced Electrocatalytic $H_2O_2$ Production

Hansong Yuan,‡[a] Yuheng Gu,‡[a] Qian Yang,‡*[a] Shun Li,[a] Jianming Zhang,[a] Long Zhang[a] and Yuqiao Zhang* [a]

[a]School of Chemistry and Chemical Engineering, Jiangsu University, Zhenjiang, Jiangsu, 212013, China. Correspondence: yuqiaozhang@ujs.edu.cn, yangqian0730@foxmail.com

‡H. Yuan, Y. Gu, and Q. Yang contributed equally to this work.

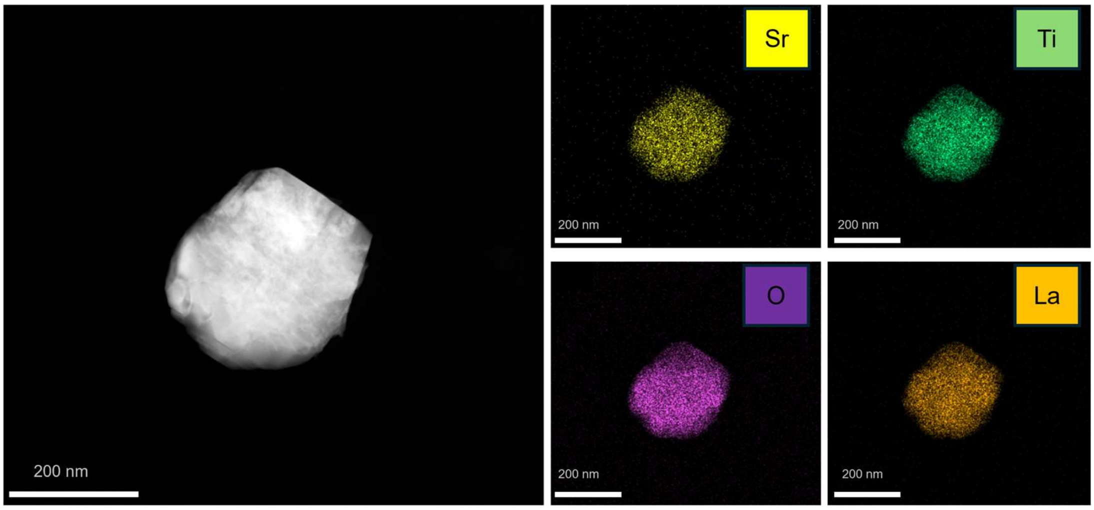


**Figure S1.** Mapping diagram of 10%$La_2O_3$-$SrTiO_3$ sample.

**Table S1.** The atomic fraction of each element

| Element | Atomic Fraction (%) |
|---|---|
| O | 59.78 |
| Ti | 18.20 |
| Sr | 18.46 |
| La | 3.56 |

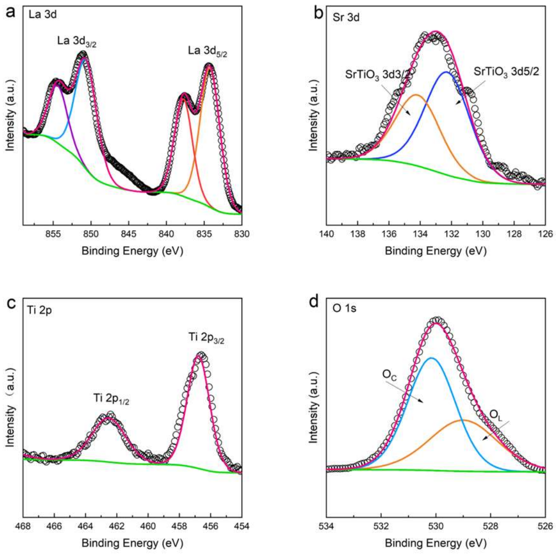


**Figure S2.** High-resolution X-ray photoelectron spectroscopy (XPS) spectra of (a) La 3d and (b) Sr 3d and (c) Ti 2p and (d) O 1s ($O_C$ and $O_L$ denote the surface-adsorbed oxygen and lattice oxygen, respectively).

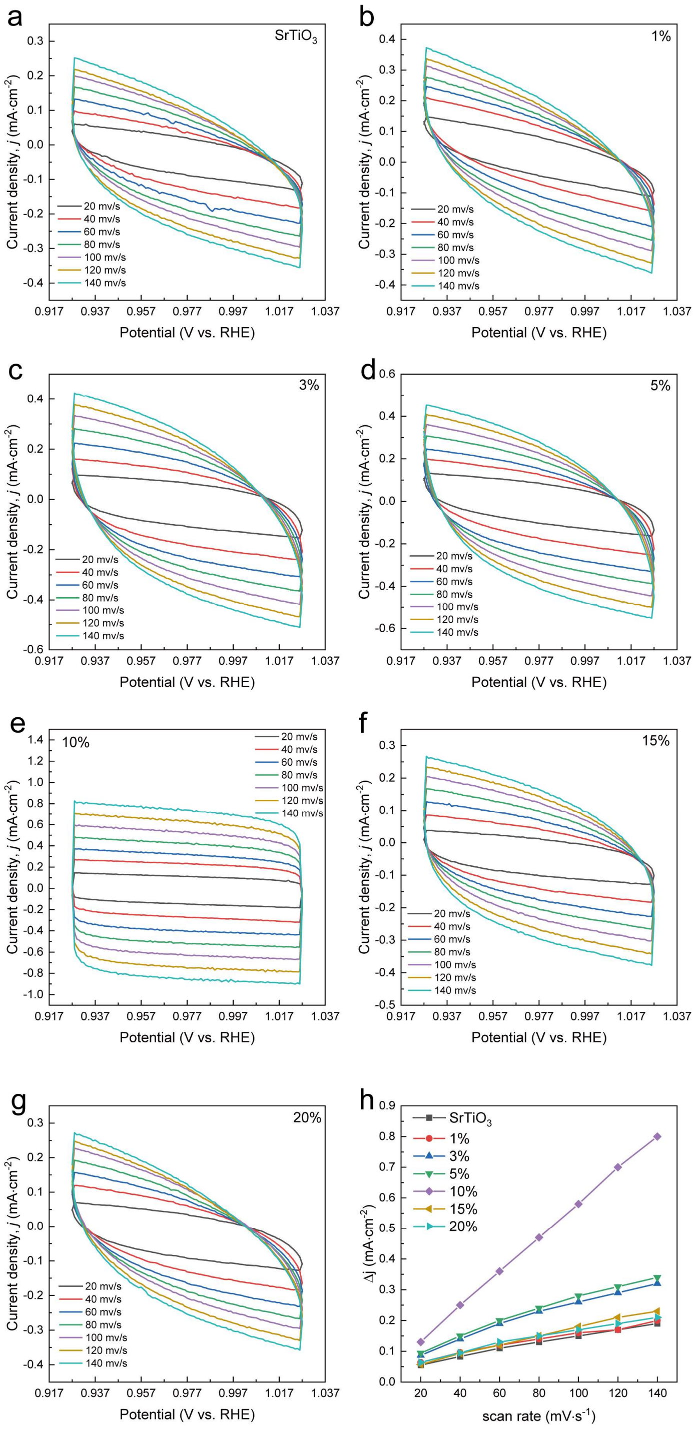


**Figure S3.** (a-g) Cyclic voltammetry curves of different electrocatalysts. (h) The corresponding electrochemically active surface area (ECSA) diagram.

**Table S2** Bilayer capacitance ($C_{dl}$) of different catalysts.

| | $SrTiO_3$ | 1% | 3% | 5% | 10% | 15% | 20% |
|---|---|---|---|---|---|---|---|
| $C_{dl}$ | 1.06 | 1.19 | 1.91 | 2.03 | 5.59 | 1.45 | 1.11 |

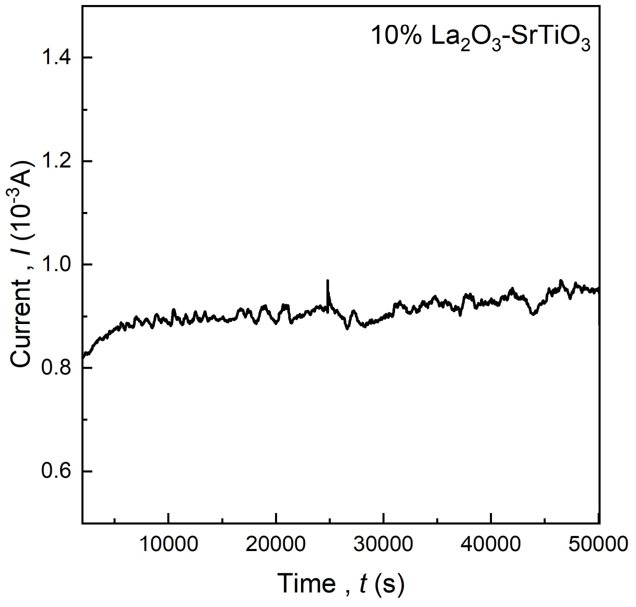


**Figure S4.** Stability testing of 10% $La_2O_3$-$SrTiO_3$ electrocatalyst.

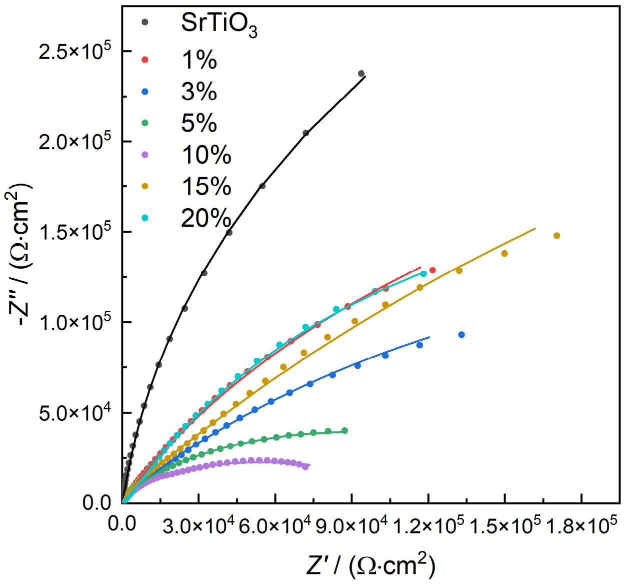


**Figure S5.** Nyquist plots of different $La_2O_3$-$SrTiO_3$ composite electrocatalysts.

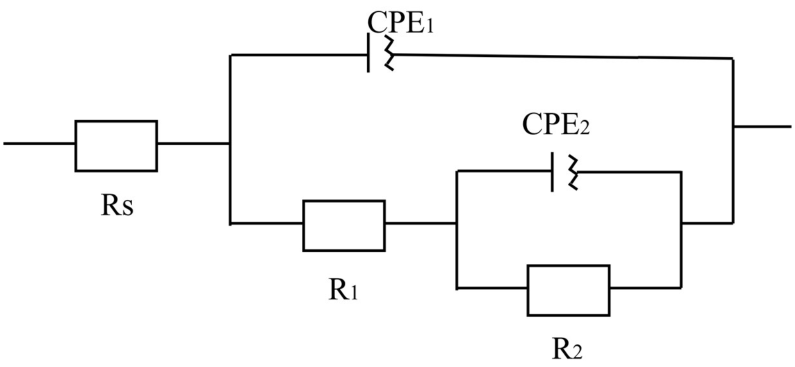


**Figure S6.** Equivalent circuit diagram for electrochemical impedance spectroscopy (EIS).

**Table S3** Electrochemical impedance spectroscopy fitting data.

| Sample | $R_s$ | $CPE_1$ | $R_1$ | $CPE_2$ | $R_2$ |
|---|---|---|---|---|---|
| $SrTiO_3$ | 37.03 | 0.0000457 | 68420 | 0.0000046 | 814540 |
| 1% | 21.85 | 0.0000171 | 5962 | 0.0000180 | 724990 |
| 3% | 21.21 | 0.0000074 | 5286 | 0.0000203 | 584150 |
| 5% | 19.81 | 0.0000090 | 1729 | 0.0000201 | 195850 |
| 10% | 22.10 | 0.0000052 | 1511 | 0.0000167 | 126530 |
| 15% | 21.83 | 0.0000078 | 5770 | 0.0000160 | 687940 |
| 20% | 18.00 | 0.0000130 | 6983 | 0.0000249 | 782210 |

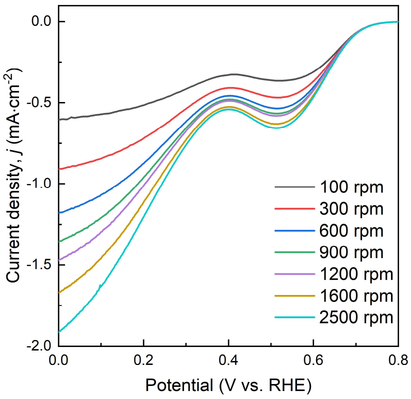


**Figure S7.** Linear sweep of voltammetry curves of the 10% $La_2O_3$-$SrTiO_3$ sample at different rotation speeds.
